# Bose glass and Fermi glass

**DOI:** 10.1038/s41598-023-39285-1

**Published:** 2023-08-01

**Authors:** Korekiyo Takahashi, Keiji Nakatsugawa, Masahito Sakoda, Yoshiko Nanao, Hiroyoshi Nobukane, Hideaki Obuse, Satoshi Tanda

**Affiliations:** 1 Department of Applied Physics, Hokkaido University, Sapporo, 060-8628 Japan; 2 Center of Education and Research for Topological Science and Technology, Hokkaido University, Sapporo, 060-8628 Japan; 3 School of Physics and Astronomy, University of St Andrews, Fife, KY16 9SS Scotland; 4 Department of Physics, Hokkaido University, Sapporo, 060-0810 Japan; 5 Research Center for Materials Nanoarchitectonics, National Institute for Material Science, Tsukuba, 305-0044 Japan; 6 Nomura Research Institute, Ltd., Tokyo, 100-0004 Japan

**Keywords:** Condensed-matter physics, Electronic properties and materials, Phase transitions and critical phenomena, Superconducting properties and materials

## Abstract

It is known that two-dimensional superconducting materials undergo a quantum phase transition from a localized state to superconductivity. When the disordered samples are cooled, bosons (Cooper pairs) are generated from Fermi glass and reach superconductivity through Bose glass. However, there has been no *universal* expression representing the transition from Fermi glass to Bose glass. Here, we discovered an experimental renormalization group flow from Fermi glass to Bose glass in terms of simple $\beta$-function analysis. To discuss the universality of this flow, we analyzed manifestly different systems, namely a Nd-based two-dimensional layered perovskite and an ultrathin Pb film. We find that all our experimental data for Fermi glass fall beautifully into the conventional self-consistent $\beta$-function. Surprisingly, however, flows perpendicular to the conventional $\beta$-function are observed in the weakly localized regime of both systems, where localization becomes even weaker. Consequently, we propose a universal transition from Bose glass to Fermi glass with the new two-dimensional critical sheet resistance close to $R_\Box = h/e^{2}$.

## Introduction

The electric conductance in the quantum localized regime (a regime where the electrical resistance increases as the temperature decreases) of two-dimensional (2D) disordered systems has been discussed in terms of Fermi glass^1–7^, i.e., Mott localization^8,9^ for strongly correlated systems and Anderson localization^10–15^ for non-interacting systems^16^, and Bose glass^17,18^. The Bose glass phase is an insulator phase with properties similar to those of Fermi glass, and can be described as the phase where the 2D bosons are localized as a result of the quenched 2D disorder. Anderson localization has been studied using $\beta$-function analysis^10,19^. Mott localization has been studied via Mott variable range hopping conduction (VRH)^20^ and via Fisher scaling for the boson Hubbard model^8,21,22^. Recently, Kapitulnik *et al*.^23^ have shown that there is an anomalous metal state that overturns the conventional wisdom in the regime below the superconducting critical sheet resistance^8,24^ $h/4e^2$ and is different from the quantum localized regime. However, a boson-fermion mixture or a transition from Fermi glass to Bose glass has not been considered in the quantum localized regime.

**Figure 1:**
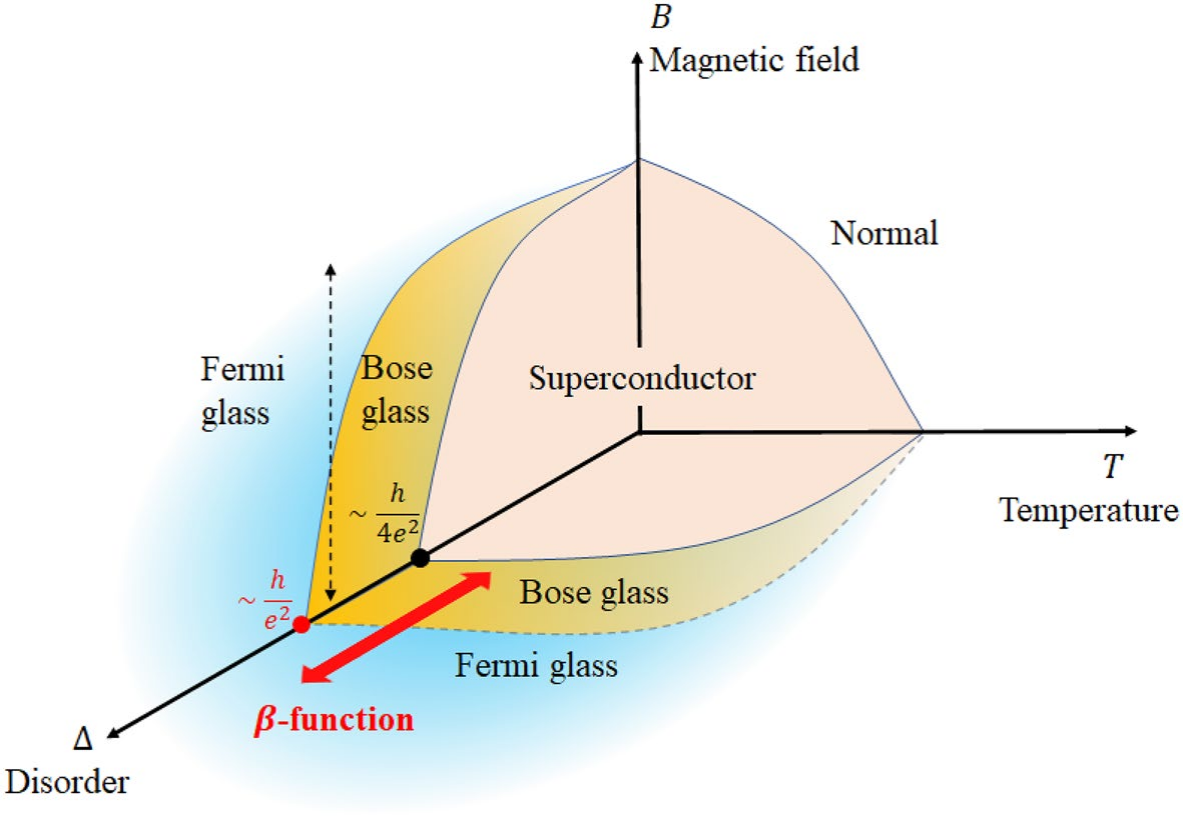
Schematic phase diagram for 2D disordered superconducting materials. The analysis in this airticle used the $\beta$-function to show the behavior of the change from Bose glass to Fermi glass before reaching superconductivity (bi-directional red arrow). The critical sheet resistance, which is the boundary between Bose glass and Fermi glass, is indicated to be around $h/e^{2}$ (red dot). The bi-directional black dotted arrow represents Paalanen et al.’s experiments^18^.

In this Article, we discovered an experimental renormalization group flow from Fermi glass to Bose glass in terms of $\beta$-function analysis. The critical sheet resistance, which is the boundary between Bose glass and Fermi glass, is indicated to be around $h/e^{2}$ as shown in Fig. 1.

**Figure 2:**
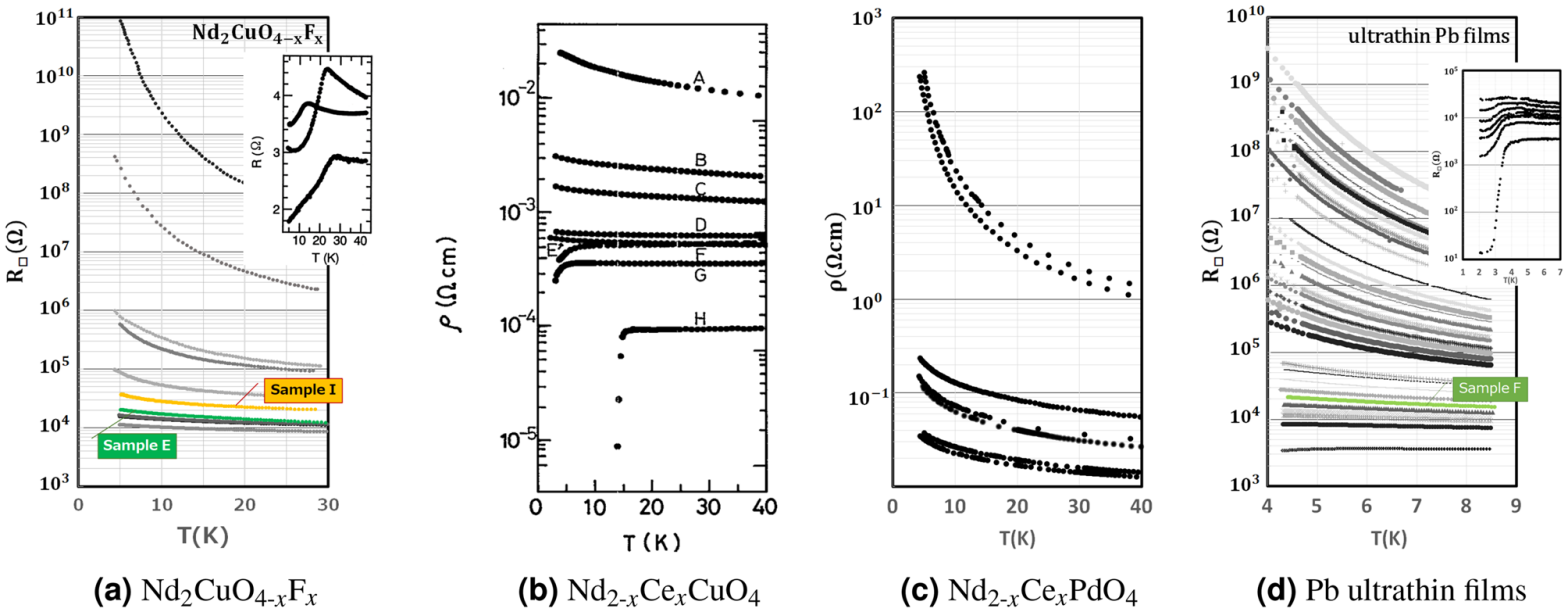
The temperature dependence of the electric resistivity (**a**) ${\textrm{Nd}}_{2}{\textrm{CuO}}_{4-x}{\textrm{F}}_{x}$^26^, (**b**) Nd_2–*x*_Ce_*x*_CuO_4_^27^, (**c**) Nd_2–*x*_Ce*x*PdO_4_^28^ and (**d**) ultrathin Pb films^29^ (thickness changed from 13.8Å  to 42.8Å). Superconductivity–insulator transition is observed in (**a**) Nd_2_CuO_4–*x*_F_*x*_ (the inset shows the superconducting state), Nd_2–*x*_Ce_*x*_CuO_4_ and (**d**) ultrathin Pb films (the inset shows the superconducting state), while in (**c**) Nd_2–*x*_Ce_*x*_PdO_4_ only the insulating state appears. Sample E of Nd_2_CuO_4–*x*_F_*x*_, Sample I of Nd_2_CuO_4–*x*_F_*x*_ and Sample F of ultrathin Pb films will be discussed in detail later. Sample E of Nd_2_CuO_4–*x*_F_*x*_ is shown to be Bose glass and Sample I of Nd_2_CuO_4–*x*_F_*x*_ is shown to be Fermi glass. Sample F of ultrathin Pb films shows a change from Fermi glass to Bose glass as the temperature becomes lower.

To discuss the universality of this flow, we analyzed manifestly different systems, namely a Nd-based two-dimensional layered perovskite and an ultrathin Pb film. The layered perovskite structure of ${\textrm{Nd}}_{2}{\textrm{CuO}}_{4}$ and ${\textrm{Nd}}_{2}{\textrm{PdO}}_{4}$ is called a $T'$-structure^25^, and it has an ideal 2D electron conductance because the conducting layer is composed of square planar units. FIG. 2 shows the temperature dependence of the electric resistance for ${\textrm{Nd}}_{2}{\textrm{CuO}}_{4-x}F_{x}$ single crystals^26^, ${\textrm{Nd}}_{2-x}{\textrm{Ce}}_{x}{\textrm{CuO}}_{4}$ thin films^27^, and ${\textrm{Nd}}_{2-x}{\textrm{Ce}}_{x}{\textrm{PdO}}_{4}$ thin films^28^. Doping causes a ${\textrm{Nd}}_{2}{\textrm{CuO}}_{4}$ system to undergo a quantum phase transition from the localized state to the superconducting state, i.e., superconductivity–insulating (S–I) transition. Superconductivity is not observed in a ${\textrm{Nd}}_{2}{\textrm{PdO}}_{4}$ system regardless of the doping amount^28^. On the other hand, the ultrathin Pb film^29^ is grown sequentially in situ by the evaporation of Pb. The film changes to a superconductor from an insulator as the film thickness increases (Fig. 2).

We find that all experimental data in Fermi glass fall beautifully into the conventional self-consistent $\beta$-function. Surprisingly, however, flows perpendicular to the conventional $\beta$-function were observed in the weakly localized regimes of both systems, where localization becomes even weaker. We show that the perpendicular flow implies the existence of Bose glass by using an experimental $\beta$-function and the temperature-derivative equation of the $\beta$-function ($\beta ^{\prime }$-function). Consequently, we can propose that there is a universal transition from Bose glass to Fermi glass with the new two-dimensional critical sheet resistance close to $R_\Box = h/e^{2}$. Since the Bose glass phase in two types of Nd-based two-dimensional layered perovskites and Pb ultrathin films both exhibit a $\beta ^{\prime }$-function different from that of the Fermi glass phase, with $R_\Box$ between $h/e^{2}$ and $h/4e^{2}$, this phenomenon is considered universal.

## Vertical flows in $\beta$-function analysis

We investigate our experimental results with $\beta$-function analysis^10,13^, which is applicable from weak to strong localization regimes. The $\beta$-function is defined as $\beta (g) \equiv \textrm{d} \ln g/\textrm{d} \ln L$, where *L* is the sample size, and *g* is the dimensionless conductance [ $g=(\hbar /e^2)\sigma$ in 2D]. The $\beta$-function shows the flow of electronic states as a function of size *L* and can be used to determine whether it is delocalized or localized, and even weakly or strongly localized. In other words, if we view the form of the $\beta$-function as a renormalization group flow, we can understand the universality of the electronic state with the scale transformation.

Experimentally, *L* is taken to be the cutoff length due to inelastic scattering: $L^2=DT^{-p}$. *D* is the diffusion constant, and *T* is the temperature. The value of exponent *p* depends on the inelastic scattering mechanism. For Nd-based 2D layered perovskite systems, $p=1.0$^26^. For ultrathin Pb films, $p=2.1$^29^. So, the $\beta$-function is derived from the experimental data of the temperature dependence of the conductance as follows:

1 $$\begin{aligned} \beta _{ {\text {EXP.}}}(g)=-\frac{2}{p} \frac{\textrm{d} \ln g}{\textrm{d} \ln T}. \end{aligned}$$

**Figure 3:**
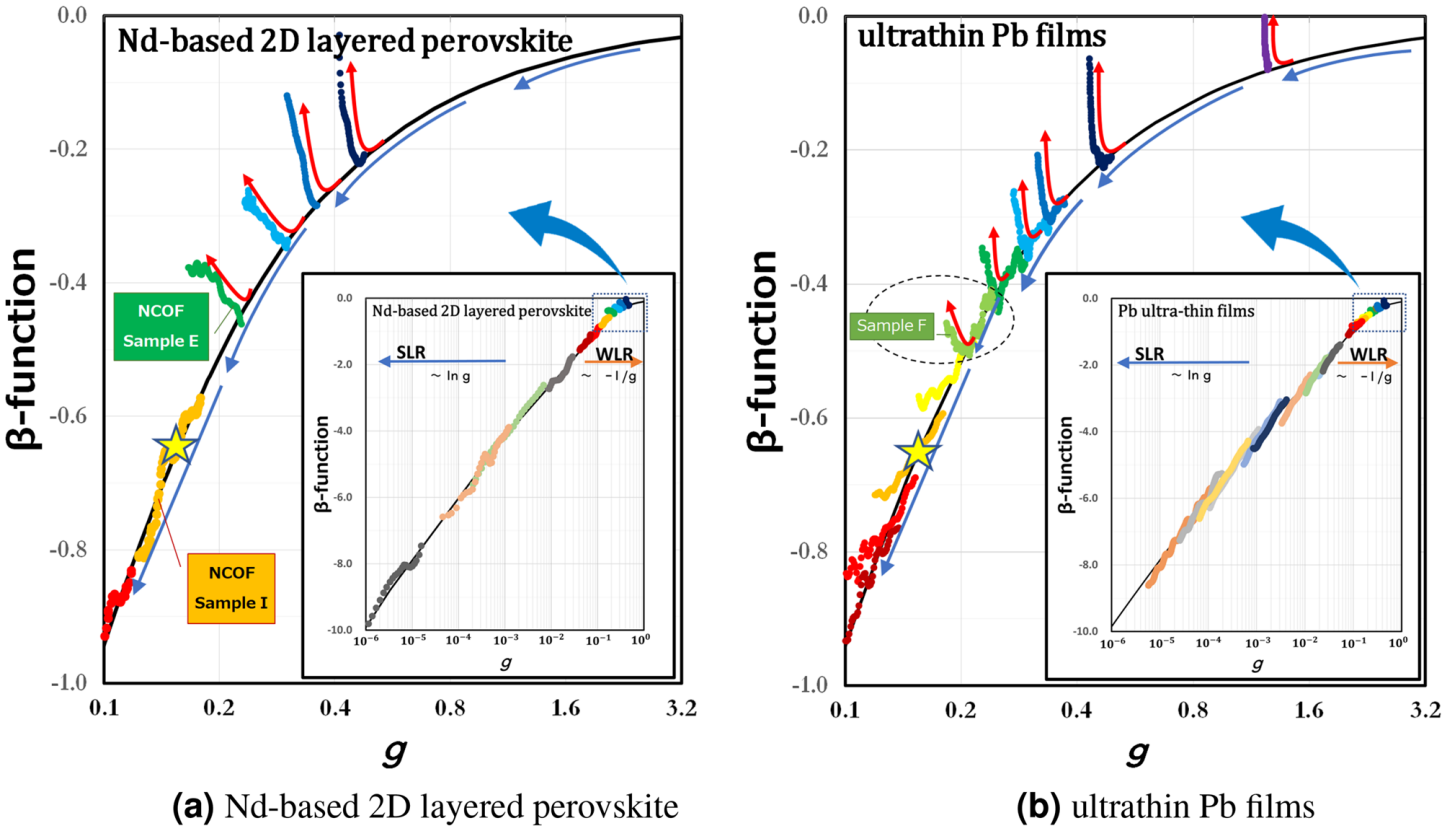
Enlarged view of $\beta _{ {\text {EXP.}}}(g)$ - the experimental $\beta$-function in a weakly localized regime. The inset shows $\beta _{ {\text {EXP.}}}(g)$ from a weak to a strong localized regime. The solid black line shows $\beta _{ {\text {VW}}}(g)$ — Vollhardt and Wölfle $\beta$-function [(eq. (5)]. The data for Nd-based 2D layered perovskite were plotted using resistance values from 5 to 25 K. The data for ultrathin Pb films were plotted using resistance values from 4 to 10 K. The color depends on the sample. If we view the $\beta$-function as a renormalization group flow, the Anderson localization $\beta$-function flow should behave like $\beta _{ {\text {VW}}}(g)$ ,as indicated with the blue arrows. However, as shown by the red arrows, we discover a different flow which is perpendicularto $\beta _{ {\text {VW}}}(g)$. In the weakly localized regime, these flows are present in both systems. In some ultrathin Pb film samples (e.g., Pb-sample-F surrounded by a dotted circle), it is clear that the flow changed from the usual Anderson localization flow to a perpendicularflow at a certain temperature. The yellow star in each graph indicates $g = 1/2\pi$ and $\beta = -2/\pi$, which is the dimensionless version of the critical sheet fermion resistance $R_\Box = h/e^{2}$. This star is the boundary value at which the perpendicularflow occurs, as determined from Fig. 5 and Appendix B. Therefore, it proves that the phenomenon of these perpendicularflows is at its least apparent when $R_\Box$ is smaller than $h/e^{2}$.

This equation is predicted to behave as follows from weak to strong localized regimes;

In a weakly localized regime, the celebrated weak localization theory in 2D^13^ predicts the logarithmic system size dependence of the dimensionless conductance as

2 $$\begin{aligned} g = g_0 - g_1 \ln \left[ \frac{L}{l} \right] , \end{aligned}$$

with a non-universal constant $g_0 = 1/(k_F l)$, where $k_F$ and *l* are the Fermi wave number and the mean free path, and a universal coefficient $g_1=1/\pi ^2$. Substituting Eq. (2) into Eq. (1) with the fact that $\ln L = - \frac{p}{2} \ln T$, we obtain the universal scaling relation as follows:

3 $$\begin{aligned} \beta _{ {\text {EXP.}}}(g) = -\frac{2}{p} \frac{\textrm{d} \ln (g_0 + g_1 \frac{p}{2} \ln T)}{\textrm{d} \ln T} = -\frac{g_{1}}{g} = -\frac{1}{\pi ^{2}g}. \end{aligned}$$

Since *p* is canceled out in the above calculation, $\beta _{ {\text {EXP.}}}(g)$ is related only to *g*. With a strong localized regime, namely the VRH theory in Mott 2D, $g \propto \exp (-\varepsilon _{ {\text {VRH}}}/T^{1/3})$, so that $\beta _{ {\text {EXP.}}}(g) \sim \ln g$. This $\varepsilon _{ {\text {VRH}}}$ is equal to the generalized activation energy^30^.

On the other hand, in the self-consistent theory of the $\beta$-function, Vollhardt and Wölfle gave a single 2D system conductivity formula for orthogonal classes from weakly to strongly localized regimes^31,32^.

4 $$\begin{aligned} g_{ {\text {VW}}}(x)=\frac{1}{2 \pi ^2}(x+1) \ln \left( \frac{1}{x^2}+1\right) \exp (-x), \end{aligned}$$

Here, $x=L/\xi \propto T^{-p/2}/\xi$ , where $\xi$ is the localization length. Furthermore, the $\beta$-function from Eq. (4) can be calculated as follows:

5 $$\begin{aligned} \beta _{ {\text {VW}}}(g)=\frac{\textrm{d} \ln g_{ {\text {VW}}}(x)}{\textrm{d} \ln x}=-\left( \frac{x^2}{x+1}+\frac{2}{\left( x^2+1\right) \ln \left( \frac{1}{x^2}+1\right) } \right) . \end{aligned}$$

We analyzed our experimental data by using the above equations: first, we estimated $\xi$ from Eq. (4). Next we described the $\beta$-function obtained from Eq. (5). Finally we overlaid the $\beta _{ {\text {EXP.}}}(g)$ data of Eq. (1) and the $\beta _{ {\text {VW}}}(g)$ data of Eq. (5), to verify whether or not they matched. We see that all the data from weakly to strongly localized regimes fall into $\beta _{ {\text {VW}}}(g)$ (inset of Fig. 3). However, if we take a closer look at the weakly localized regime, as shown in Fig. 3, we discover that the data exhibit different *perpendicular flows* (indicated by the red arrows) against the usual Anderson localization $\beta$-function curve (indicated by the blue arrows). In the weakly localized regime, these perpendicularflows are present in both Nd-based 2D layered perovskite and ultrathin Pb films. In some ultrathin Pb film samples, it is clear that the flow changes from the usual Anderson localization flow to the perpendicularflow at a certain temperature (e.g., Pb-sample-F surrounded by a dotted circle). Nd_2_CuO_4–*x*_F_*x*_ single crystals and Nd_2–*x*_Ce_*x*_CuO_4_ thin films have different perpendicularflows, but Nd_2–*x*_Ce_*x*_PdO_4_ does not have them. It is considered to be a phenomenon that is a precursor to the transition from weak localization to superconductivity. The discovery of the upturn from the $\beta _{ {\text {VW}}}(g)$ to the perpendicularflow suggests that the single parameter hypothesis is not sucient^33–36^. However, this $\beta$-function analysis method will suggest us to understand the causes and the conditions of these perpendicularflows.

**Figure 4:**
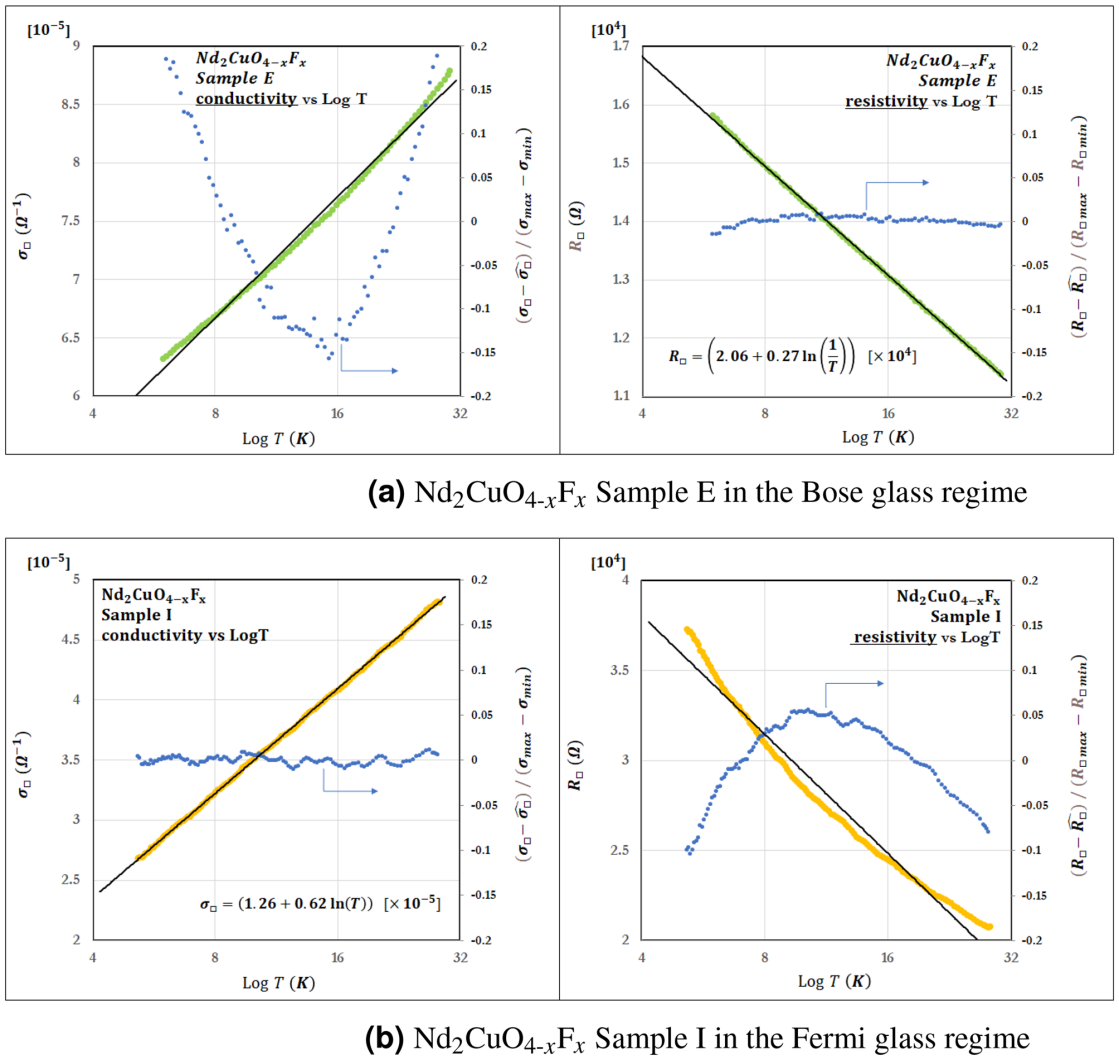
Graphs comparing the $\log T$ dependence of $\sigma _\Box$ (left) and $R_\Box$ (right). We analyzed two samples before and after the perpendicular flow occurred; namely (**a**) Nd_2_CuO_4–*x*_F_*x*_ (NCOF) sample E in which this perpendicular flow appears and (**b**) NCOF sample I in which the perpendicular flow does not appear. (NCOF-sample-E is along the red arrow, and NCOF-sample-I is along the blue arrow in Fig. 3). The solid black line shows the regression line. The left vertical axis of the graph is $\sigma _\Box$ or $R_\Box$. The graph’s horizontal axis is $\log T$, and the right vertical axis is the value obtained by subtracting $\hat{\sigma _\Box }$ or $\hat{R_\Box }$ from the experimental value. For standardization, we divide by the value obtained by subtracting the minimum value from the maximum value of the experimental data on the vertical axis. Clearly, NCOF-sample-E is more suitable for the $\log T$ dependence of the resistivity than that of the conductivity, while NCOF-sample-I is the opposite. The perpendicular flow in the $\beta$-function indicates $\rho \sim \ln (1/T)$ rather than $\sigma \sim \ln T$. As a result, we find a difference between the $\log T$ dependence of the conductivity and resistivity. Appendix C shows graphs comparing the $\log T$ dependence of $\sigma _\Box$ and $R_\Box$ on other data.

To understand the mechanism of perpendicular flows, we again analyze the temperature dependence of resistivity in more significant detail. The functional forms of these perpendicular flows appear in the weakly localized regime but are not adapted to $-1/g$. Therefore, we analyzed two samples before and after the perpendicular flow occurred; namely (a) Nd_2_CuO_4–*x*_F_*x*_ (NCOF) sample E in which this perpendicular flow appears and (b) NCOF sample I in which the perpendicular flow does not appear. As a result, we find a difference in the temperature dependence of conductivity $\sigma$ and resistivity $\rho (= 1 / \sigma )$. As shown in Fig. 4, the $\ln T$ dependence of the NCOF-sample-E resistivity is more suitable than that of the conductivity. In contrast, according to the Andeson localization theory, the NCOF-sample-I conductivity has $\ln T$ dependence. Although these are in the same weakly localized regime, the perpendicular flow is clearly characterized by $\rho \sim \ln (1/T)$ rather than by $\sigma \sim \ln T$.

Das et al.^37^ pointed out that the weak localization of bosons occurring on the insulator side of the S–I transition in 2D is characterized by $\rho \sim \ln (1/T)$. Therefore, the state of the sample with the perpendicular flow can be regarded as boson localization or the Bose glass phase. These experimental results indicate that the Bose glass regime has been found.

## The transition from Fermi glass to Bose glass

**Figure 5:**
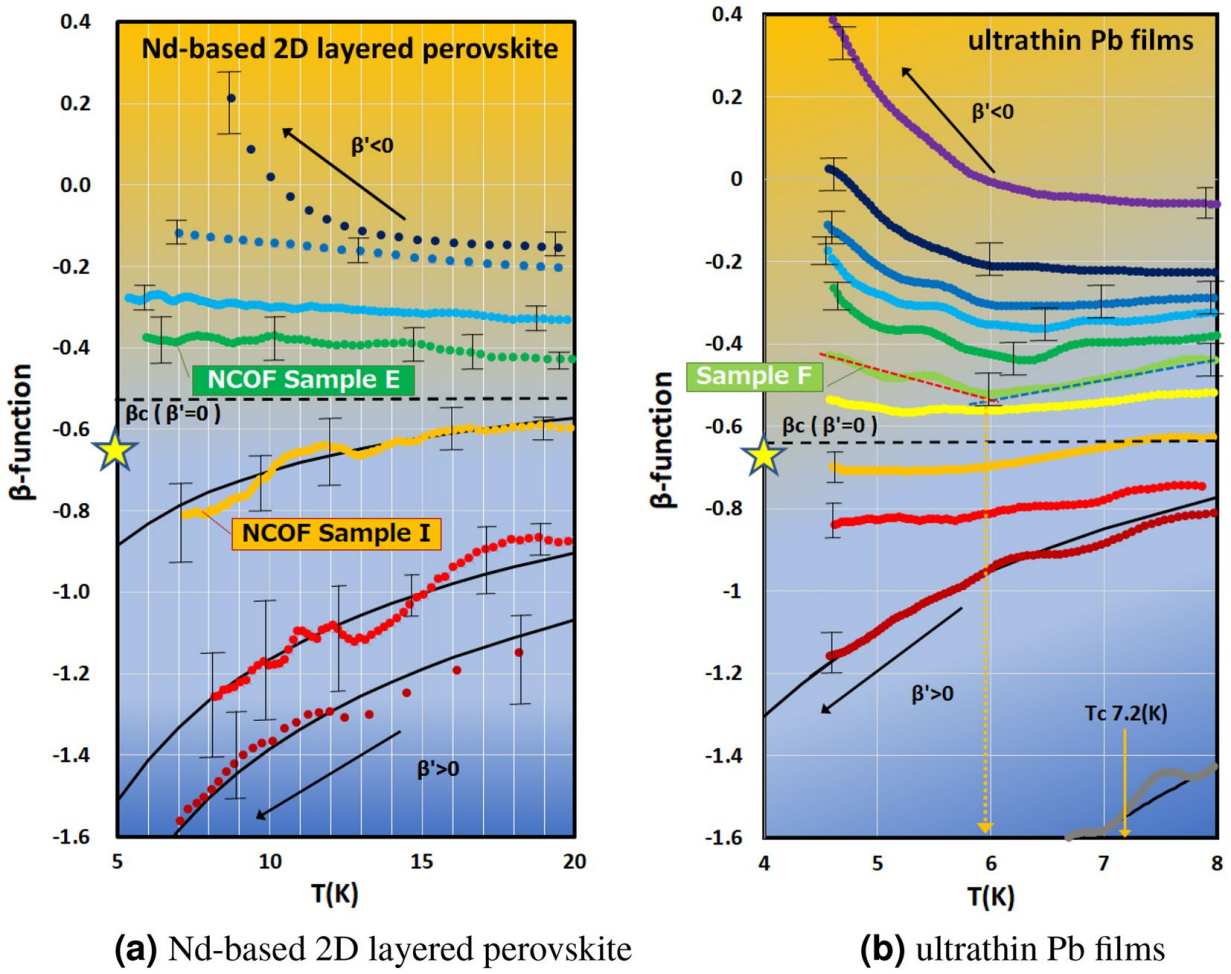
Graphs of the relationship between the value of $\beta _{ {\text {EXP.}}}(g)$ and temperature *T* with a series of experimental data in a weakly localized regime. Interestingly, the temperature dependence of $\beta _{ {\text {EXP.}}}(g)$ is very similar to the S–I transition graph. As the localization of each sample weakens (from the bottom to the top of the graph), the slope of each set of data changes continuously from positive to negative in both (**a**) Nd-based 2D layered perovskite and (**b**) ultrathin Pb films. The color depends on the sample. The solid black line shows $\beta _{ {\text {VW}}}(g)$—Vollhardt and Wölfle $\beta$-function [Eq. (5)]. We can see that among the weakly localized samples, those with strong localization ($\beta _{ {\text {EXP.}}}^{\prime }(g) > 0$) fit into $\beta _{ {\text {VW}}}(g)$. However, in the (**b**) graph, as the localization becomes weaker, the slope of Pb sample F changes from positive (blue dotted line) to negative (red dotted line) when the temperature falls below around 6 K. Since the superconducting transition temperature $T_c$ of Pb (in a clean system) is around 7.2 K, it is conceivable that a boson appears. We also investigated the critical value of $\beta _{ {\text {EXP.}}}(g)$, denoted as $\beta _{ {\text {C}}}$ when the slope of $\beta _{ {\text {EXP.}}}^{\prime }(g)$ goes to zero. $\beta _{ {\text {C}}}$ was obtained by using the slope and the intercept of the vertical axis for each sample at a specific low temperature. We obtained $\beta _{ {\text {C}}} =-0.6 \pm 0.1$ in these two different types of samples. This value is almost the same 0.64 ($\simeq 2/\pi$) as shown by the yellow star, and has a value of $g=1/2\pi$ when converted using Eq. (3). This value is the dimensionless version of the critical sheet fermion resistance $R_\Box = h/e^{2}$.

Next, to investigate the change condition from Bose glass to Fermi glass, we plotted the temperature dependence of $\beta _{ {\text {EXP.}}}(g)$ instead of the resistance, as shown in Fig. 5 with a series of experimental data. Interestingly, the temperature dependence of $\beta _{ {\text {EXP.}}}(g)$ in the weakly localized regime is very similar to the S–I transition graph. As the localization of each sample weakens (from the bottom to the top of Fig. 5), the slope of each set of data changes continuously from positive to negative in both Nd-based 2D layered perovskite and ultrathin Pb films.

To understand the change in the slope of $\beta _{ {\text {EXP.}}}(g)$ flows in Fig. 5, we use the temperature-derivative equation of the $\beta$-function shown below,

6 $$\begin{aligned} {\beta _{ {\text {EXP.}}}^{\prime }(g)= - \frac{\textrm{d} \ln |\beta _{ {\text {EXP.}}}(g)|}{\textrm{d} \ln T}.} \end{aligned}$$

The change in the $\beta$-function flow should appear in the positive or negative sign of $\beta _{ {\text {EXP.}}}^{\prime }(g)$. Substituting the two equations $\sigma =\sigma _0 + \sigma _1 \ln T$ and $\sigma = 1/\rho =1/(\rho _0+\rho _1\ln (1/T))$ ($\sigma , \sigma _0, \sigma _1, \rho _0, \rho _1> 0$) we see that the signs are different as follows.

7 $$\begin{aligned} \beta _{ {\text {EXP.}}}^{\prime }(g)= & {} \frac{\sigma _1}{\sigma _0+\sigma _1 \ln T}>0, \qquad \quad (\sigma =\sigma _0 + \sigma _1 \ln T) \end{aligned}$$

8 $$\begin{aligned} \beta _{ {\text {EXP.}}}^{\prime }(g)= & {} -\frac{\rho _1}{\rho _0+\rho _1 \ln (1/T)}<0, \quad (\sigma = \frac{1}{\rho } =\frac{1}{\rho _0+\rho _1\ln (1/T)}). \end{aligned}$$

In the above calculations, we treat $\sigma$ as *g*. While the $\sigma$ has a dimensionality, this does not change the sign of $\beta _{ {\text {EXP.}}}^{\prime }(g)$. These calculation results confirm that the slope of $\beta _{ {\text {EXP.}}}(g)$ reflects the temperature-dependent functional form of the conductivity and resistivity. To check this, we analyzed all the data for $\beta _{ {\text {EXP.}}}^{\prime }(g)<0$ and found that they were more suitable for $\rho \sim \ln (1/T)$ than $\sigma \sim \ln T$. However, in the ultrathin Pb film data in Fig. 5b, there were samples where the sign of $\beta _{ {\text {EXP.}}}^{\prime }(g)$ changed from positive to negative when the temperature decreased. For Pb-sample-F, the sign of $\beta _{ {\text {EXP.}}}^{\prime }(g)$ changed around 6 K. Since the superconducting transition temperature $T_c$ of Pb (in the clean system) is around 7.2 K, it is conceivable that bosons are formed. On the other hand, NCOF-sample-E did not exhibit any change of sign. This may be attributed to the measurement temperature range. Another set of data measured up to the high temperature regime was analyzed, and the change was confirmed below the superconducting transition temperature $T_c$ of the Nd_2_Pd_1–*x*_Cu_*x*_O_4–*x*_F_*x*_ (see Appendix A).

We also investigated the critical value of $\beta _{ {\text {EXP.}}}(g)$, denoted as $\beta _{ {\text {C}}}$, when the slope of $\beta _{ {\text {EXP.}}}^{\prime }(g)$ went to zero. From Fig. 5, $\beta _{ {\text {C}}}$ was obtained by using the slope and the intercept of the vertical axis for each sample at a specific low temperature. We obtained $\beta _{ {\text {C}}} =-0.6 \pm 0.1$ with these two different types of samples (see Appendix B). This value is almost equal to $-0.64(\simeq -2/\pi )$ and becomes the value of $g=1/2\pi$, which we can convert to $\beta =-2/\pi$ by using Eq. (3). The critical *g* does not indicate $R_\Box = h/4e^{2}$ of the superconducting critical sheet resistance, but is at least close to $R_\Box = h/e^{2}$ of the critical sheet fermion resistance. Mapping these values in Fig. 3 as a yellow star, we can see that the phenomenon of these perpendicular flows occurs at least when *g* is larger than $1/2\pi$. This $g=1/2\pi$ value is transformed into the dimensionless version of the critical sheet fermion resistance $R_\Box = h/e^{2}$. Based on the above results, at least two conditions are required to generate Bose glass; $\beta _{ {\text {EXP.}}}^{\prime }(g) < 0$ and $R_\Box < h/e^{2}$. The point at which the sign of $\beta _{ {\text {EXP.}}}^{\prime }(g)$ changes is related to the superconducting transition temperature $T_c$. This experimental analysis regarding the $\beta$-function clearly demonstrated the criteria for the Fermi glass to Bose glass transition. In other words, it proved the existence of boson formation even in a localized regime.

## Discussion

Finally, we suggest that the fermion-only theory may be insufficient to explain the weakly localized regime just before reaching superconductivity in *2D disordered* superconducting materials. Fisher’s theory is also insufficient because it is based on boson-only or fermion-only in the strongly localized regime represented by VRH. Meanwhile, disordered thin-film superconductors in the weakly multifractal regime can exhibit a S–I transition and allow for a boson-fermion mixture^38–44^. The experiment using a disordered superconductor TiN revealed the existence of Cooper pairs above the superconducting transition temperature $T_c$^45^. Therefore, the boson–fermion mixture state and boson-only state in the weakly localized regime are important areas in clarifying the relationship between statistics (fermion, boson, and anyon) and localization. We discover an experimental renormalization group flow from Fermi glass to Bose glass by employing $\beta$-function analysis. To discuss the universality of Bose glass flow, we analyzed distinctively different systems, namely a Nd-based two-dimensional layered perovskite and an ultrathin Pb film. What these two systems have in common is that they are both 2D disordered systems, but the difference is that a Nd-based 2D layered perovskite is disordered by doping while an ultrathin Pb film is structurally disordered. Furthermore, unlike an ultrathin Pb film, a Nd-based 2D layered perovskite is a strongly correlated electron system^9,46^. Although these two systems may have different superconductivity mechanisms, the flow of the experimental $\beta$-function exhibits the same behavior. The analysis results obtained for the two systems in common are shown in Table 1.

**Table 1:**
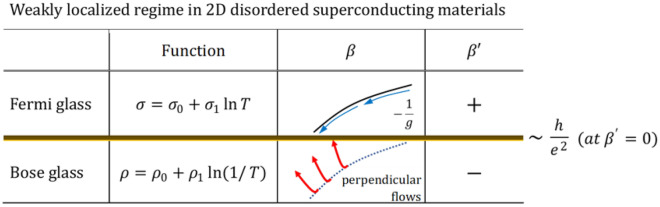
Summary of the experimental results.

In the weakly localized regime where 2D Anderson localization dominates, both Fermi glass and Bose glass exist. The existence of the Bose glass regime is clearly shown by the experimental $\beta$-function and the sign of the $\beta ^\prime$-function (the temperature derivative of the experimental $\beta$-function). We propose a universal transition from Bose glass to Fermi glass with the new two-dimensional critical sheet resistance close to $R_\Box = h/e^{2}$ at $\beta ^\prime = 0$.

These results suggest the following mechanism of the crossover. With varying disorders in the weakly localized regime, Anderson localization ($\sigma \sim \ln T$) is observed when $R_\Box$ is larger than $h/e^{2}$. When $R_\Box$ is smaller than $h/e^{2}$, bosons and vortices are generated and the state becomes Bose glass. Furthermore, fermions disappear when $R_\Box$ is smaller than $h/4e^{2}$, and superconductivity occurs. It seems reasonable that Bose glass appears between $h/e^{2}$ and $h/4e^{2}$.

Paalanen et al.^18^ identified Bose glass as the regime where the Hall resistance $\rho _{xy}$ has a zero or a finite value when the longitudinal resistance $\rho _{xx}$ diverges at low temperatures and in a certain magnetic field (the bi-directional black dotted arrow in Fig. 1). On the other hand, we used the experimental $\beta$-function to identify the flow perpendicular to the 2D Anderson localized flow as Bose glass (the bi-directional red arrow in Fig. 1). This analysis constitutes a simple method of identifying weak boson/fermion localization. As an application of these results, even if superconductivity does not appear in a 2D material, the discovery of Bose glass flow in its $\beta$-function may provide a hint that could allow us to make the material superconductive by changing the disorder parameters.

## Supplementary Information

Supplementary Information.

## Data Availability

The datasets generated during and/or analyzed during the current study are available from the corresponding author on reasonable request.
