# Supplementary material for: Bose glass and Fermi glass
Source: Sci Rep. 2023 Aug 1;13:12434. doi: 10.1038/s41598-023-39285-1 (PMC10394042; doi:10.1038/s41598-023-39285-1)
Supplement: Supplementary file 1 — Supplementary Information. [file 41598_2023_39285_MOESM1_ESM.pdf]

Supplementary Information : Bose Glass and Fermi Glass

Korekiyo Takahashi[1,2,6,*], Keiji Nakatsugawa[2,5,+], Masahito Sakoda[1,2], Yoshiko Nanao[3], Hiroyoshi Nobukane[4,2], Hideaki Obuse[1,2,+], and Satoshi Tanda[1,2,+]

[1]Department of Applied Physics, Hokkaido University, Sapporo, 060-8628, Japan
[2]Center of Education and Research for Topological Science and Technology, Hokkaido University, Sapporo, 060-8628, Japan
[3]School of Physics and Astronomy, University of St Andrews, Fife, KY16 9SS, Scotland
[4]Department of Physics, Hokkaido University, Sapporo, 060-0810, Japan
[5]International Center for Materials Nanoarchitectonics,National Institute for Material Science, Tsukuba, 305-0044 Japan
[6]Nomura Research Institute, Ltd., Tokyo, 100-0004, Japan
[*]korere@gmail.com
[+]these authors contributed equally to this work

ABSTRACT

This PDF file includes:
Supplementary Text (Appendix A, Appendix B and Appendix C)
Figure A1, Figure B1 ,Figure C1 and Figure C2

Appendix A The relationship between $\beta_{\text{EXP.}}(g)$ and temperature T with $Nd_2Pd_{1\text{-}x}Cu_xO_{4\text{-}y}F_y$

We analyzed special substances in $Nd_2Pd_{1\text{-}x}Cu_xO_{4\text{-}y}F_y$ films grown on CaF_2. The Nd-based high-temperature superconductor Nd_2CuO_4 develops superconductivity as a result of appropriate reduction treatment. We investigated the temperature dependence of the resistance of $Nd_2Pd_{1\text{-}x}Cu_xO_4$ in which this Cu was replaced with Pd (Figure 1, left panel). If the proportion of Cu is large ($x = 0.84$), it is expected that bosons will be generated at a certain temperature and the $\beta_{\text{EXP.}}(g)$ slope will change. The analysis result is shown on the right in Figure 1. At about 20K (red arrow) the experimental data moved away from $\beta_{\text{vw}}(g)$ and there was a positive and negative change in $\beta'_{\text{EXP.}}(g)$. This temperature is almost the same as the critical temperature T_c of the Nd-based high-temperature superconductor. Figure 1 indicates the transition from Fermi glass (yellow dots) to Bose glass (green dots) on Nd-based 2D layered perovskite as well as ultrathin Pb films.

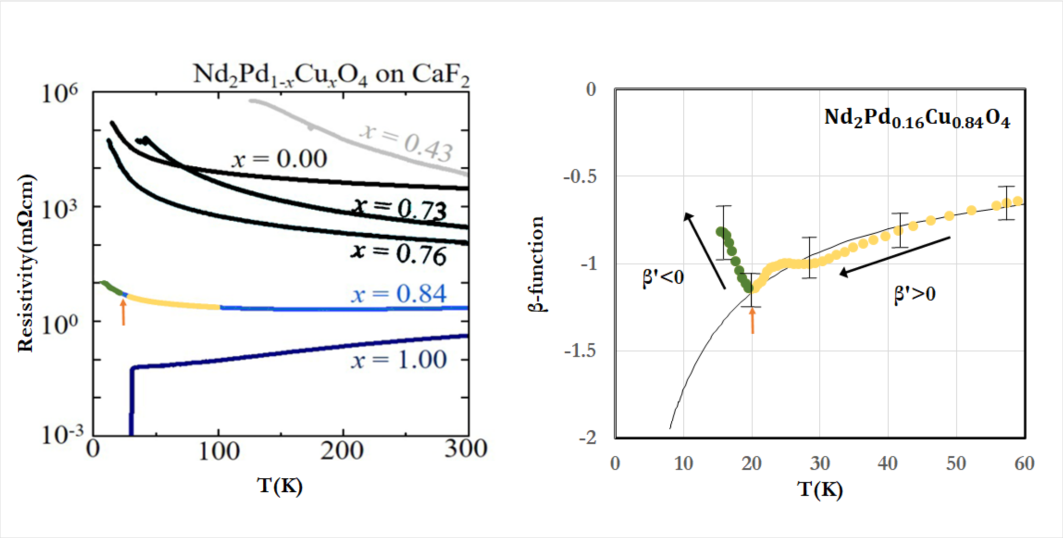

Figure A1. The left panel shows the temperature dependence of the resistance of $Nd_2Pd_{1\text{-}x}Cu_xO_{4\text{-}y}F_y$ films grown on CaF_2 ($x = 0.43 \sim 1.00$). The right panel shows the relationship between $\beta_{\text{EXP.}}(g)$ and temperature T with $Nd_2Pd_{0.16}Cu_{0.84}O_4$. This sample was the closest to the superconducting state among the localized samples, . To calculate $\beta_{\text{EXP.}}(g)$ requires the dependence of the value of exponent p on the mechanism of inelastic scattering, but since it was not measured, it was calculated as $p = 1.5$. The black solid line shows $\beta_{\text{vw}}(g)$. It was at about 20K (red arrow) that the experimental data moved away from $\beta_{\text{vw}}(g)$ and the sign of $\beta'_{\text{EXP.}}(g)$ changed. This temperature is almost the same as the critical temperature T_c of the Nd-based high-temperature superconductor. No change in $\beta'_{\text{EXP.}}(g)$ was observed in samples other than $x = 0.84$. This graph indicates the transition from Fermi glass (yellow dots) to Bose glass (green dots) as the temperature decreases.

Appendix B Boundary value when $\beta'_{\text{EXP.}}(g) = 0$ is in the weakly localized regime

So, if there is a boundary between positive and negative $\beta'_{\text{EXP.}}(g)$ in the weakly localized regime, when $\beta'_{\text{EXP.}}(g) = 0$, what is the value of $\beta_{\text{EXP.}}(g)$ and the conductivity g? For each graph in Figure 5, we regarded $\beta_{\text{EXP.}}(g)$ as a linear function of T in a particular temperature range such as $\beta_{\text{EXP.}}(g) = aT + b$ (where a is the slope of $\beta_{\text{EXP.}}(g)$ and b is the intercept of $\beta_{\text{EXP.}}(g)$). Figure 1 shows a graph in which a and b of each graph in Figure 5 are mapped, and the $\beta_{\text{EXP.}}(g)$ value at T = 0 is obtained. From the experimental data in Figure 1, we obtained $\beta_c = -0.6 \pm 0.1$ in these two different types of samples. This value is almost equal to 0.64($= 2/\pi$) and becomes the value of $g = 1/2\pi$ to which we can convert $\beta = -2/\pi$ using eq.(2). This g is a dimensionless version of the critical sheet fermion resistance $R_\square = h/e^2$. The boundary of $\beta'_{\text{EXP.}}(g) = 0$ separates the Bose glass and Fermi glass regimes. In other words, this result reveals that the condition for the occurrence of weakly localized $\rho \sim \ln(1/T)$ is $g > 1/2\pi$. This means that superconductivity cannot occur above $R_\square = h/e^2$, so the results of these analyses are consistent.

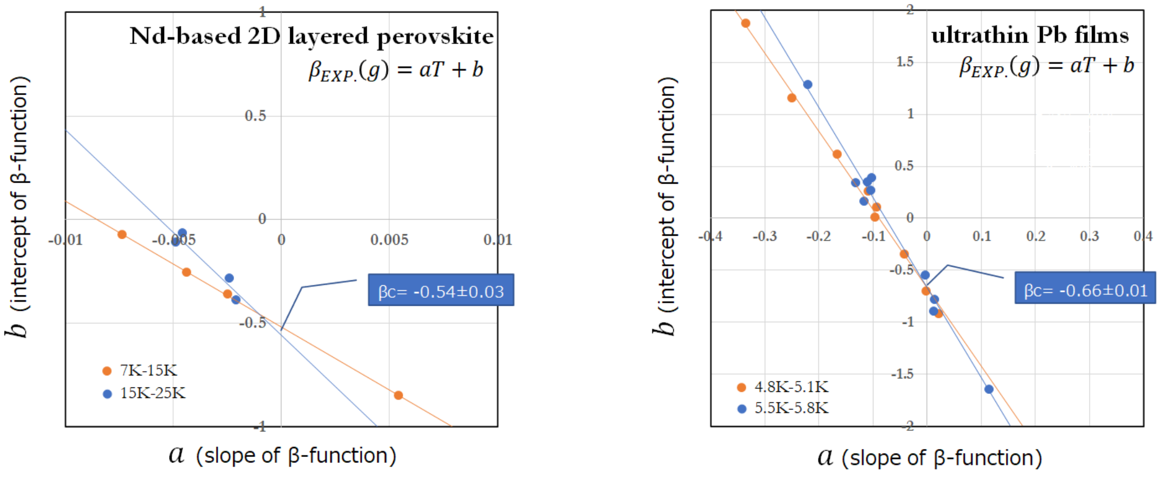

(a) Nd-based 2D layered perovskite systems

(b) ultrathin Pb films

Figure B1. The above graphs are the analysis to find a boundary value when $\beta'_{\text{EXP.}}(g) = 0$ in the weakly localized regime. For each graph in Figure 5, we regarded $\beta_{\text{EXP.}}(g)$ as a linear function of T in a particular temperature range such as $\beta_{\text{EXP.}}(g) = aT + b$. The horizontal axis a is the slope of $\beta_{\text{EXP.}}(g)$ and the vertical axis b is the intercept of $\beta_{\text{EXP.}}(g)$. These graphs showed a and b of each graph in Figure 5, and the $\beta_{\text{EXP.}}(g)$ value at $T = 0$ was obtained. From the experimental data obtained for the Nd-based 2D layered perovskite systems, we obtained $\beta_{\text{EXP.}}(g) = -0.54 \pm 0.03$. And from the experimental data for the ultrathin Pb films, we obtained $\beta_{\text{EXP.}}(g) = -0.66 \pm 0.01$. From the experimental data in Figure 1, we obtained $\beta_c = -0.6 \pm 0.1$ in these two different types of samples. This value is almost equal to 0.64($\simeq 2/\pi$) and becomes a value of $g = 1/2\pi$, to which we can convert $\beta = -2/\pi$ using eq.(2). This g is the dimensionless version of the critical sheet fermion resistance $R_\square = h/e^2$. The boundary of $\beta'_{\text{EXP.}}(g) = 0$ separates the Bose glass and Fermi glass regimes.

Appendix C Graphs comparing the $\log T$ dependence of $\sigma_{\square}$ and $R_{\square}$ on other data

By using the β-function as a function of T, we could investigate the changing condition from Bose glass to Fermi glass as shown in Figure. 1. We discussed the $\log T$ dependence of $\sigma_{\square}$ and $R_{\square}$ using NCOF Sample E and NCOF Sample I near the Bose glass and Fermi glass boundary ($\beta' = 0$) in the main text. In order to highlight the difference in $\log T$ dependence, we analyzed NCOF Sample L and NCOF Sample N away from the boundary (See Figure.2).

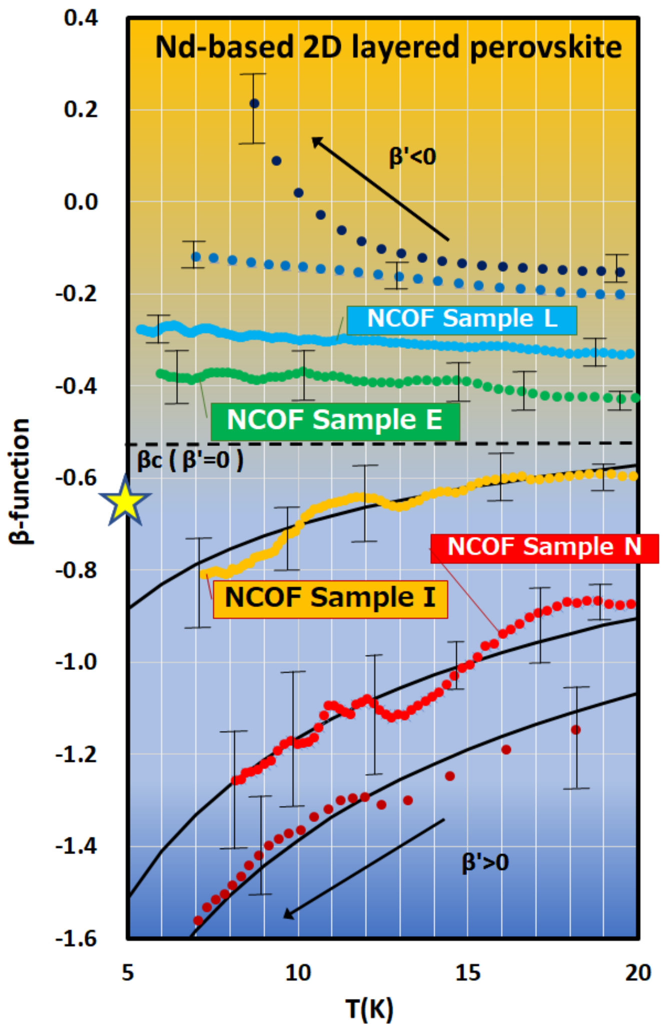

Figure C1. Nd-based 2D layered perovskite's graph of the relationship between the value of $\beta_{\mathrm{EXP.}}(g)$ and temperature T with a series of experimental data in a weakly localized regime. In the main text, we discussed $\log T$ dependence of $\sigma_{\square}$ and $R_{\square}$ using NCOF Sample E and NCOF Sample I near the Bose glass and Fermi glass boundary ($\beta' = 0$). To highlight the difference in $\log T$ dependence, we analyzed NCOF Sample L (light blue color) and NCOF Sample N (red color) away from the boundary.

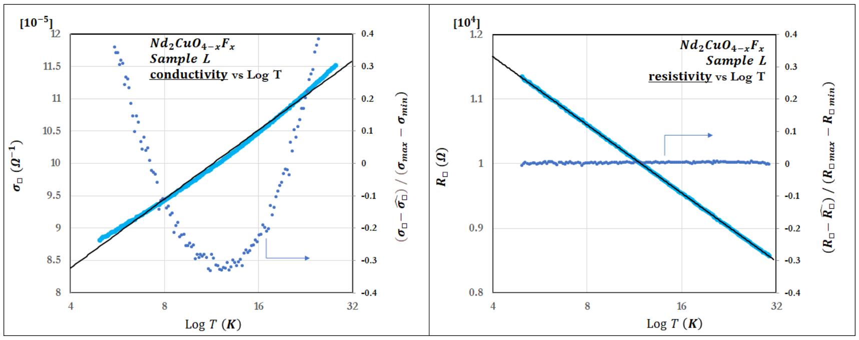

(a) $Nd_2CuO_{4\text{-}x}F_x$ Sample L in the Bose glass regime

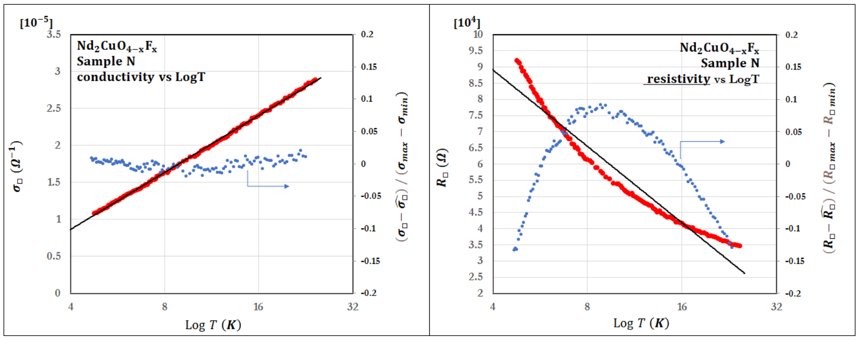

(b) $Nd_2CuO_{4\text{-}x}F_x$ Sample N in the Fermi glass regime

Figure C2. Graphs comparing the $\log T$ dependence of $\sigma_\square$ (left) and $R_\square$ (right). (a) $Nd_2CuO_{4\text{-}x}F_x$ (NCOF) sample L in which this perpendicular flow appears and (b) NCOF sample N in which the perpendicular flow does not appear in the β-function. The solid black line shows the regression line. The left vertical axis of the graph is $\sigma_\square$ or $R_\square$. The graphs horizontal axis is $\log T$, and the right vertical axis is the value obtained by subtossoverracting $\hat{\sigma_\square}$ or $\hat{R_\square}$ from the experimental value. For standardization, we divide by the value obtained by subtracting the minimum value from the maximum value of the experimental data on the vertical axis. Clearly, NCOF-sample-L is more suitable for the $\log T$ dependence of the resistivity than that of the conductivity, while NCOF-sample-N is the opposite. The perpendicular flow in the β-function indicates $\rho \sim \ln(1/T)$ rather than $\sigma \sim \ln T$. As a result, we find a difference between the $\log T$ dependence of the conductivity and resistivity in other data in the main text.

Data availability

The datasets generated during and/or analysed during the current study are available from the corresponding author on reasonable request.
